# Home Heart Hospital Associated With Reduced Hospitalizations and Costs Among High‐Cost Patients With Cardiovascular Disease

**DOI:** 10.1002/clc.24302

**Published:** 2024-06-14

**Authors:** Michael Shen, Kareem Osman, Daniel M. Blumenthal, Kaelin DeMuth, Yixiang Liu

**Affiliations:** ^1^ Novolink Health (Previously Duxlink Health) A Division of Cardiovascular Associates of America Sunrise Florida USA; ^2^ University of California Los Angeles David Geffen School of Medicine, Department of Medicine Los Angeles California USA; ^3^ Novocardia A Division of Cardiovascular Associates of America Celebration Florida USA; ^4^ Cardiology Division Massachusetts General Hospital Boston Massachusetts USA; ^5^ Harvard Medical School Boston Massachusetts USA; ^6^ Philadelphia College of Osteopathic Medicine South Georgia Moultrie Georgia USA

**Keywords:** cost, digital health, healthcare utilization, high need high cost, hospital at home, readmission, remote patient monitoring, telemedicine

## Abstract

**Background:**

There is no widely accepted care model for managing high‐need, high‐cost (HNHC) patients. We hypothesized that a Home Heart Hospital (H3), which provides longitudinal, hospital‐level at‐home care, would improve care quality and reduce costs for HNHC patients with cardiovascular disease (CVD).

**Objective:**

To evaluate associations between enrollment in H3, which provides longitudinal, hospital‐level at‐home care, care quality, and costs for HNHC patients with CVD.

**Methods:**

This retrospective within‐subject cohort study used insurance claims and electronic health records data to evaluate unadjusted and adjusted annualized hospitalization rates, total costs of care, part A costs, and mortality rates before, during, and following H3.

**Results:**

Ninety‐four patients were enrolled in H3 between February 2019 and October 2021. Patients' mean age was 75 years and 50% were female. Common comorbidities included congestive heart failure (50%), atrial fibrillation (37%), coronary artery disease (44%). Relative to pre‐enrollment, enrollment in H3 was associated with significant reductions in annualized hospitalization rates (absolute reduction (AR): 2.4 hospitalizations/year, 95% confidence interval [95% CI]: −0.8, −4.0; *p* < 0.001; total costs of care (AR: −$56 990, 95% CI: −$105 170, −$8810; *p* < 0.05; and part A costs (AR: −$78 210, 95% CI: −$114 770, −$41 640; *p* < 0.001). Annualized post‐H3 total costs and part A costs were significantly lower than pre‐enrollment costs (total costs of care: −$113 510, 95% CI: −$151 340, −$65 320; *p* < 0.001; part A costs: −$84 480, 95% CI: −$121 040, −$47 920; *p* < 0.001).

**Conclusions:**

Longitudinal home‐based care models hold promise for improving quality and reducing healthcare spending for HNHC patients with CVD.

## Graphical abstract

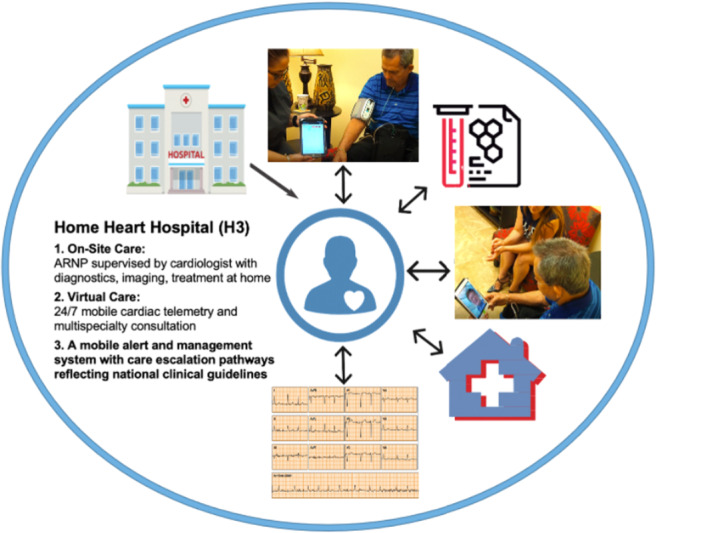

**Abbreviations**
ADMIT: annualized number of admissions per patient
ALOS: annualized length of stay in patient (in days)
ARR: absolute risk reduction
BPCI: bundled payment for care improvement
CC: conventional care
CMS: Center for Medicare and Medicaid Services
COST: mean annualized total costs of care per patient for each period
ECG: electrocardiogram
H3: Home Heart Hospital
HaH: hospital at home
HNHC: high‐need, high‐cost
ICU: intensive care unit
NNT: number needed to treat
NP: nurse practitioner
Part A COST: mean annualized part A spending per patient for each period
PCP: primary care physician
SCCM: Society of Critical Care Medicine
SDU: step‐down unit

## 1 Background

Current population health management approaches fail to meet the medical and financial challenges posed by high‐need, high‐cost (HNHC) patients, who comprise approximately 5% of the US population 18 years or older, and account for as much as 50% of total US health care spending [1]. Factors impacting this disparity are patient heterogeneity and unpredictability due to incurable, highly complex late‐stage diseases creating higher risk for development of acute medical problems and rapid clinical deterioration [2, 3, 4].

Several programs launched over the past three decades, including the Center for Medicare and Medicaid Services' (CMS) Bundled Payment for Care Improvement (BPCI), Ambulatory Intensive Care and the Camden Coalition's hotspotting program, have attempted to improve care quality and reduce health care spending among HNHC patients [5, 6]. These initiatives have yielded inconsistent results, and studies suggest that any significant improvements in clinical outcomes and spending are likely small in magnitude [5, 6]. In November 2020, the CMS launched the hospital at home (HaH) program in an effort to reduce demand for limited hospital beds during the COVID‐19 pandemic. Published research has found associations between HaH and improved care quality, including lower rates of complications, and lower healthcare spending [7, 8, 9, 10]. While HaH may represent a viable approach for more efficiently managing acute illnesses among HNHC patients, evidence‐based care models for longitudinal ambulatory management of HNHC patients are lacking and sorely needed.

**Figure 1:**
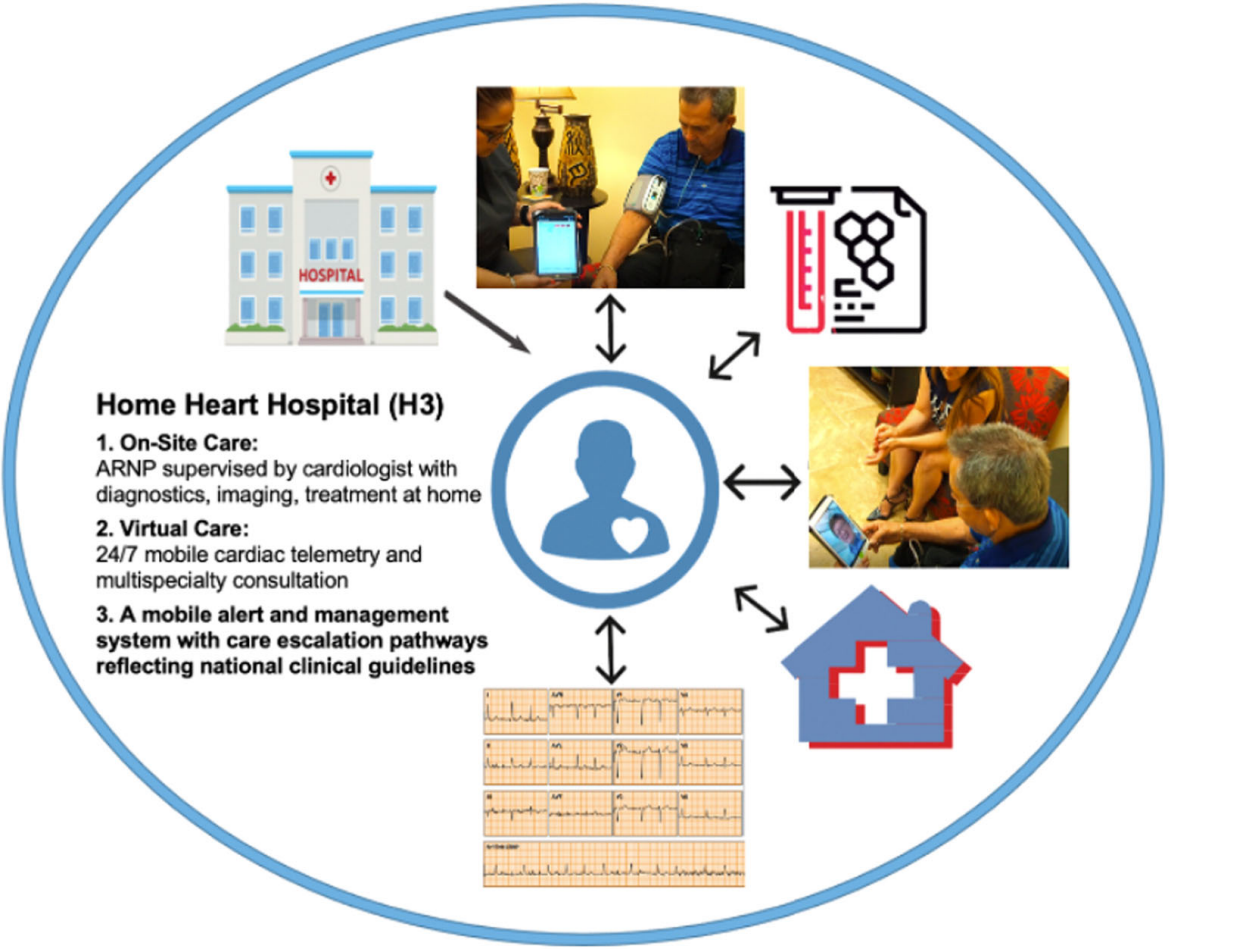
The H3 care model is designed to provide acute care services in the patient's home, including cardiac telemetry, which is utilized at the discretion of the treating cardiologist. The included photos display the use of 4G‐connected devices as part of the remote patient monitoring bundle of sensors provided to each patient.

In 2019 Novolink Health launched Home Heart Hospital (H3), an extended‐length (i.e., 60–90 day episode) HaH model managed by a multidisciplinary team of specialist physicians, in three counties in Southeast Florida (Miami‐Dade, Broward, and Palm Beach). This care model provides access to 24 h‐a‐day home‐based monitoring (electrocardiography, telemetry, blood pressure, weight, and pulse oximetry) and telehealth‐based clinician access, regular in‐home visits from a nurse practitioner (NP), and in‐home treatment of acute and chronic conditions (Figure 1). Most patients were admitted for primary cardiac diagnoses, including coronary artery disease, arrhythmias, and congestive heart failure. Initial evaluations of this program demonstrate that it is associated with improvements in quality of care and lower total costs of care for a broad population of patients [11, 12, 13]. We hypothesized that enrollment in H3 would be associated with lower health care utilization and costs of care for HNHC patients with advanced cardiac conditions.

## 2 Methods

We evaluated outcomes of interest using a retrospective within‐subjects cohort design. All patients or their healthcare proxies formally consented to allow their health information to be used for publication and research purposes.

### 2.1 The H3 Model

The H3 model includes in‐home care and caregiver education from a NP supervised by a cardiology physician and supported with resources to perform diagnostic labs and imaging (X‐ray, ultrasound) in the home, and treatment delivery including oral and intravenous medications, oxygen, nebulizers, and other commonly performed hospital treatments. Virtual care consisted of multispecialty (cardiology, infectious disease, nephrology, and pulmonology) consultations as needed as well as continuous (24/7) access to personal 12‐lead ECG and real‐time mobile cardiac telemetry monitoring, similar to that available in a cardiac step‐down or unit (SDU) or intensive care unit (ICU). The H3 care team was capable of responding in real‐time to highly morbid cardiovascular problems and managing them in the home.

Novolink Health has developed customizable processes and protocols to evaluate and escalate care as needed, including a mobile alert and management system to allow 24/7 monitoring and response to urgent needs. These protocols reflected published clinical guidelines for the management of acute cardiovascular conditions and complaints—including for heart failure, arrhythmias, chest pain, and recognition of myocardial infarction and are integrated into the electronic medical record.

Three levels of care were available to H3 enrollees: Level 0: standard medical‐surgical ward care; Level 1: Level 0 services plus continuous telemetry; and Level 2: SDU/ICU level of care based on Society of Critical Care Medicine (SCCM) criteria [14]. The specialist‐led team was able to treat patients on noninvasive ventilators, percutaneous gastrostomy tube feeds, and hemodialysis in the home setting.

A home visit by the treating physician or NP visit initiated the H3 care episode (starting date). After episode initiation, the NP provided acute care services, including intravenous infusions, imaging, phlebotomy, wound care, and the provision of durable medical equipment. The level of care needed for each H3 enrollee was determined by the Novolink Health team in consultation with the patient's primary care physician (PCP) and using the SCCM criteria. Tablets, SIM cards, blood pressure machines, pulse oximeters, weight scales, and compact, portable 12‐lead ECG were provided to all patients and were utilized as the clinical team deemed necessary. H3 care episodes lasted until a patient's clinical needs were met and s/he was discharged from H3, transferred to a higher level of care, declined further treatment, was admitted to hospice, or died. To ensure care continuity, the H3 care model includes robust discharge education and a coordinated hand‐off process with the patient's primary care physician (PCP).

#### 2.1.1 Study Population

Eligibility criteria for H3 included: age >18 years, at‐least three chronic medical problems, including at least one cardiovascular disease, at least two hospitalizations in the prior 12 months for which cardiovascular disease was deemed by the PCP to be an important contributing factor, and provision of informed consent personally or via a healthcare proxy. Patients with cardiopulmonary emergencies at the time of referral (i.e., cerebrovascular accidents or acute myocardial infarction), with hemodynamic instability from sepsis or respiratory distress/failure, or already enrolled in hospice were not eligible for H3. Most patients were referred to H3 from their PCP before discharge from the hospital, with some patients referred upon their postdischarge follow‐up appointment.

#### 2.1.2 Time Periods

Outcomes were evaluated by grouping data into three time periods: (1) Conventional care (CC), which included 180 days before H3 enrollment; (2) H3 enrollment, which began with enrollment and ended on the date of discharge; and (3) the post‐H3 period, which included the 180 days following H3 discharge. The post‐H3 period was subdivided into 30‐, 90‐, and 180‐day periods to create standardized time periods during which to evaluate for changes in care and spending patterns following discharge from H3.

#### 2.1.3 Data

Data on patient demographics, clinical comorbidities were obtained from electronic medical record chart abstraction. For 71/94 patients, who were insured by a Humana Health Maintenance Organization (Humana was the primary insurance carrier for all study patients) data on hospitalizations, inpatient length of stay, and Medicare Part A/B/D cost for the CC, H3, and post‐H3 enrollment periods was extracted from Medicare claims. Cost data included the costs of billed H3 services. Data on hospitalizations and inpatient length of stay for the remaining 23 patients were directly obtained from the electronic medical record for the CC and H3 periods. Post‐H3 and cost data was not obtained for these 23 patients given lack of access to paid claims.

Clinical and demographic covariates included: age; sex; race/ethnicity (black, Hispanic/Latino, white); area deprivation index by analysis of home zip code [15], diagnosis of congestive heart failure, atrial fibrillation, acute coronary syndrome, coronary artery disease, cancer, chronic obstructive pulmonary disease, chronic kidney disease (stage 2 or greater), cerebrovascular accident, diabetes mellitus, infection (history of osteomyelitis, pneumonia, sepsis, diverticulitis, pyelonephritis, urinary tract infection, or chronic cellulitis), cirrhosis, or a mental health condition (defined as major depression, bipolar affective disorder, posttraumatic stress disorder, generalized anxiety disorder, or schizophrenia).

#### 2.1.4 Outcomes

Primary outcomes included the average number of hospitalizations per patient per period and the average number of inpatient days per patient period, which were annualized to adjust for varying lengths of time spent in each time period (referred to as ADMIT and ALOS, respectively). Secondary cost outcomes included mean annualized total costs of care per patient for each period (referred to as COST); and mean annualized part A spending per patient for each period (referred to as Part A COST).

#### 2.1.5 Analysis

For demographic and clinical characteristics, nominal and ordinal variables were presented as total counts with percentages of the total, and continuous variables were presented as means and standard deviations from the mean.

Primary and secondary outcomes were calculated at the patient and population level for each time period. We performed unadjusted comparisons for each outcome across the CC, H3, and post‐H3 time periods. To control for differing lengths of time spent in the CC, H3, and post‐H3 periods, we calculated annualized rates of each outcome and repeated the comparisons. Data for patients still enrolled in H3 at conclusion of the study period were excluded from the analysis of post‐H3 outcomes. Patients who died during H3 were not included in analyses of post‐H3 outcomes. We also calculated absolute differences in annualized rates of the primary and secondary outcomes between CC and H3, CC and post‐H3, and H3 and post‐H3 periods. Absolute risk reduction (ARR) and number needed to treat (NNT) were calculated for each time period as well.

We also constructed a mixed‐effects model to account for the excluded post‐H3 period outcomes data for individuals who were still in H3 at the time of study completion, which we used to compare our primary and secondary outcomes. Furthermore, we performed a sensitivity analysis evaluating ADMIT, ALOS, and COST for the 71 patients who were discharged from H3 during the study enrollment period. Post‐hoc Tukey's multiple comparisons tests were performed for statistical hypothesis testing for difference in ADMIT, ALOS, COST, and Medicare Part A COST between time periods. A Greenhouse‐Geisser correction was used if the estimated epsilon was less than 0.75. Significance was set at *p* < 0.05. The software used for this analysis was GraphPad Prism 8.0.

## 3 Results

Between February 2019 and October 2021, 94 patients were admitted to H3. H3 enrollees averaged 75 years of age; 48 (51%) were female, 66 (70%) were white, 7 (7%) were black, and 22 (23%) were Hispanic (Table 1). On average, enrollees had three medical comorbidities (SD: 1.6); 47 patients (50%) had congestive heart failure, 35 (37%) had atrial fibrillation, and 41 (44%) suffered from coronary artery disease (41, 44%). The average area deprivation index among enrollees was 47. The level of care distribution of enrollees in this study included 35 patients with med‐surg level of care (SCCM level 0), 54 patients with telemetry (SCCM level 1), and five patients at intermediate care/step‐down level of care (SCCM level 2). Patients spent an average of 80 days (95% confidence interval [95% CI]: 67, 92) enrolled in H3. The CC and post‐H3 periods were each 180 days long (Table 2).

**Table 1:** Characteristics of patients enrolled in H3.

Characteristic	Patients (*N* = 94)
Age, mean (SD)	75 (12)
Female, *N* (%)	48 (51)
Congestive heart failure, *N* (%)	47 (50)
Atrial fibrillation, *N* (%)	35 (37)
Acute coronary syndrome, *N* (%)	12 (13)
Coronary artery disease, *N* (%)	41 (44)
Cancer, *N* (%)	6 (6)
Chronic obstructive pulmonary disease, *N* (%)	15 (16)
Chronic kidney disease, *N* (%)	31 (33)
Cerebrovascular accident, *N* (%)	40 (43)
Diabetes mellitus, *N* (%)	41 (44)
Infection,^a^ *N* (%)	19 (20)
Cirrhosis, *N* (%)	3 (3)
Number of medical comorbidities,^b^ mean (SD)	3 (1.6)
Mental health condition,^c^ *N* (%)	21 (22)
Race/ethnicity, *N* (%)	
Black	6 (6)
White	66 (70)
Hispanic/Latino	22 (23)
Average area deprivation index, mean (SD)^d^	47 (13)
Ever smoker, *N* (%)	27 (29)
BMI (SD)	28 (7)

*Note:* Values are mean (SD) or *n* (%).

^a^ Infection was defined as history of osteomyelitis, pneumonia, sepsis, diverticulitis, pyelonephritis, urinary tract infection, or chronic cellulitis.

^b^ Medical comorbidities were defined as those conditions represented in Table 1.

^c^ Mental health conditions were defined as major depressive disorder, bipolar disorder, posttraumatic stress disorder, generalized anxiety disorder, or schizophrenia.

^d^ The Area Deprivation Index was used to evaluate the level of socioeconomic advantage of each patient, and was evaluated using each patient's home zip‐code [12].

**Table 2:** Unadjusted utilization outcomes before, during, and following enrollment in H3.

	CC	H3 (*n* = 94)	30 days post‐H3^a^ (*n* = 71)	90 days post‐H3^a^ (*n* = 71)	180 days post‐H3^a^ (*n* = 71)
Days in period (95% CI)	180	80 (67–92)**	30	90	180
Total admission count per period	174	37	3	15	31
Total inpatient days per period	1,209	183	14	86	185
Mean admissions per person (95% CI)	2.0 (1.8–2.2)	0.4 (0.3–0.5)***	0.04 (0.01–0.07)***	0.2 (0.1–0.4)***	1.3 (0.4–1.0)*
Mean inpatient days per person (95% CI)	12.9 (10.7–14.0)	2.0 (1.3–2.6)***	0.2 (0.1–0.3)***	1.2 (0.3–0.9^)^ ***	5.2 (4.3–6.1)*

*Note:* The CC period is the reference period, and all statistical comparisons are relative to this period. CC: 180 day period preceding enrollment in the H3 program (*n* = 94). H3: period enrolled in the H3 program (*n* = 94).

^a^ Days post‐H3: Represents outcomes during 30‐, 90, and 180‐day periods after discharge from H3 program.

*** *p* < 0.001

** *p* < 0.01

* *p* < 0.05.

### 3.1 Level of Care Distribution and Mortality

There was a trend of a direct relationship between level of care and length of enrollment in H3 (*p* = 0.06). Five patients died while enrolled in H3 (5.3% mortality rate). Two deaths occurred during unavoidable hospital admissions—one inpatient death was due to sepsis from a wound infection following coronary artery bypass graft surgery; the second inpatient death resulted from cardiac arrest due to cocaine overdose. Three deaths were among patients referred to hospice. There was no significant difference in ADMIT or mortality across levels of care (Supporting Information S1: Table 1).

### 3.2 Admissions and Inpatient Days

During CC, ADMIT was 4.2 per patient year, which decreased to 1.9 per year during H3, corresponding to 2.4 fewer admissions per patient year, on average, among patients in H3 (95% CI for absolute difference between H3 and CC periods: −0.8, −4.0; *p* < 0.01; Tables 3 and 4) and a 57% lower ARR for hospitalization (NNT: 1.8 to prevent one hospitalization). During the post‐H3 period, ADMIT was 2.8, which was not significantly different compared to the H3 period (95% CI for absolute difference in ADMIT between post‐H3 and H3 periods: −0.7, 2.6).

**Table 3:** Annualized admissions, inpatient days, and health care costs before, during, and following enrollment in H3.

	CC	H3	30 Days Post H3^a^	90 Days Post H3^a^	180 Days Post H3^a^
Mean annualized admissions per person (95% CI)^b^	4.2 (3.2–5.3)	1.9 (1.0–2.7)**	0.5 (0.2–0.8)***	0.8 (0.4–1.6)***	2.8 (1.8–3.9)
Mean annualized number of inpatient days per person (95% CI)^b^	27.4 (18.6–36.1)	7.3 (3.9–10.8)***	2.5 (1.3–3.8)***	4.8 (1.2–3.6)***	10.6 (5.7–15.5)**
Mean annualized total costs of care per person, $ (95% CI)^c^	$164 510 ($124 490, $204 530)	$107 520 ($82 720, $132 310)*	$11 980 ($8210, $15 570)***	$25 800 ($19 820, $31 780)***	$51 000 ($34 570, $67 430)***
Mean annualized Part A spending per person, $ (95% CI)^c^	$102 340 ($67 740–$136 940)	$24 140 ($12 010–$36 260)***	$3890 ($2980, $4800)***	$10 320 ($7810, $12 830)***	$17 860 ($8500–$27 220)***

*Note:* Values are *n* (95% CI) or *n* (% of total). The CC period is the reference period, and all statistical comparisons are relative to this period. The mixed effects model included both random effects and fixed effects, including missing data, intersubject variability, and time period.

^a^ Days Post = H3: Represents outcomes during 30‐, 90, and 180‐day periods after discharge from H3 program.

^b^ Numbers for each period: CC: *N* = 94; H3: *N* = 94; Post‐H3: *N* = 71.

^c^ Numbers for each period: CC: *N* = 71; H3: *N* = 71; Post‐H3: *N* = 71.

*** *p* < 0.001

** *p* < 0.01

* *p* < 0.05.

**Table 4:** Absolute difference in admissions, inpatient days, and health care costs 180 days before, during, and 180 days following enrollment in H3.

	H3*‐*CC^a^	Post‐H3‐CC^a^^,^ ^b^	Post‐H3‐H3^b^
Absolute difference in annualized admissions (ADMIT) per person (95% CI)^c^	−2.4 (−0.8, −4.0)**	−1.4 (−3.2, 0.4)^n.s.^	0.9 (−0.7, 2.6)^n.s.^
Absolute difference in annualized inpatient days (ALOS) per person (95% CI)^c^	−20.0 (−31.5, −8.6)***	−16.8 (−29.0, −4.5)**	3.3 (−4.0, 10.6)^n.s.^
Absolute difference in annualized total costs of care (COST) per person (95% CI)	−$56 990 (−$105 170, −$8810)*	−$113 510 (−$151 340, −$65 320)***	−$56 520 (−$104 700, −$8330)*
Absolute difference in annualized Part A spending (Part A COST) per person (95% CI)^d^	‐$78 210 (−$114 770, −$41 640)***	−$84 480 (−$121 040, −$47 920)***	$−6280 (−$42 840, $30 290)^n.s.^

Abbreviation: n.s. = not significant.

^a^ CC period: 180 days before H3 enrollment.

^b^ Post‐H3 period: 180 days after discharge from H3.

^c^ Patient volume for each category: CC: *N* = 94; H3: *N* = 94; Post‐H3: *N* = 71.

^d^ Patient volume for each category: CC: *N* = 71; H3: *N* = 71; Post‐H3: *N* = 71.

*** *p* < 0.001

** *p* < 0.01

* *p* < 0.05.

Compared to the CC period, enrollment in H3 was associated with a 20 day decrease in ALOS per patient year (95% CI for absolute difference in ALOS between H3 and CC periods: −31.5, −8.6; *p* < 0.001) (Table 4). Transitioning from H3 into the post‐H3 period was not associated with a significant change in ALOS (95% CI for absolute difference in ALOS between H3 and post‐H3 periods: −4.0, 10.6).

In the mixed effects models, there was a significant effect on ADMIT (*F*(2.0, 242.5) = 6.21, *p* < 0.01) and ALOS (*F*(2.0, 152) = 8.93, *p* < 0.001) based on time categorization (Supporting Information S1: Figure 1). H3 was associated with significant reductions in ADMIT (*q*(93) = 3.53, *p* < 0.01) and ALOS (*q*(93) = 4.23, *p* < 0.001). The post‐H3 period also showed a significant decrease in ALOS from CC (*q*(70) = 3.31, *p* < 0.01). Modest differences in these outcomes between the H3 and post‐H3 periods were observed, but were not statistically significant (Supporting Information S1: Figure 1).

In a sensitivity analysis including only the 71 patients who were insured by the health maintenance organization who completed 180 days of post‐H3 followup, both ADMIT and ALOS were similar to the group of 94 patients in the primary analysis (Supporting Information S1: Table 2).

#### 3.2.1 Healthcare Spending

Costs data were available for 71 of 94 patients. Mean COST per patient were $164 510 (95% CI: $124 490, $204 530) during CC, $107 520 (95% CI: $82 720, $132 310) during H3, and $51 000 ($34 570, $67 430) during post‐H3 (Table 3). On average, part A COST accounted for 62% of COST during CC, 22% during H3, and 35% during post‐H3 (Supporting Information S1: Figure 2).

Enrolling in H3 was associated with $56 990 and $78 210 reductions in COST and Part A COST, respectively (95% CI for absolute difference in COST: −$105 170, −$8810; *p* < 0.05; 95% CI for absolute difference in Part A COST: −$114 770, −$41 640; *p* < 0.001). In addition, during the post‐H3 period, COST and Part A COST remained $113 510 and $84 480 lower than during the CC period (95% CI for absolute difference in COST between post‐H3 and CC periods: (−$151 340, −$65 320; *p* < 0.001; 95% CI for absolute difference in Part A COST: −$121 040, −$47 920; *p* < 0.001) (Table 4).

In the mixed effects model, COST varied significantly between the CC, H3, and post‐H3 periods (*F*(2, 210) = 13.07, *p* ≤ 0.001). Specifically, COST was significantly lower during H3 than before the intervention (*q*(70) = 3.95, *p* < 0.05), and significantly lower during the post‐H3 period as compared to the CC, or pre‐intervention, period (*q*(70) = 7.86, *p* < 0.001) (Table 4).

The mixed effects model showed a significant effect of time period on Part A COST (*F*(2, 210) = 12.59, *p* < 0.001). Post‐hoc Tukey's multiple comparisons tests showed a significant difference in Part A COST between CC and H3 (*q*(70) = 7.14, *p* ≤ 0.001) and Part A COST remained significantly lower in the post‐H3 period, as compared to the CC period (*q*(70) = 7.71, *p* < 0.001) (Table 4).

## 4 Discussion

In this study, we show that enrolling HNHC patients with cardiovascular comorbidities in an intensive longitudinal home‐based care model is associated with significant and clinically and financially meaningful reductions in hospitalization rates, inpatient days, total costs of care, and part A spending. Collectively, these data suggest that models like H3 hold promise for improving health outcomes, reducing hospital‐based healthcare utilization, and reducing health expenditures for medically complex patients with substantial healthcare utilization. Additionally, we have identified a potential lasting benefit with reduced utilization outcomes and cost for patients after discharge from H3, raising the possibility that the H3 intervention produces clinical benefits extending beyond H3 enrollment—this hypothesis represents a direction for future studies.

The rates and patterns of mortality observed in this study—including 5% observed mortality rate during H3, with zero deaths at home, two deaths in the inpatient setting, and three deaths among patients referred to hospice—is comparable in magnitude and pattern previously published estimates of inpatient outcomes among HNHC populations, which have estimated in‐hospital mortality at 3%. Moreover, these mortality rates are quite favorable in comparison to published 30‐ and 90‐day postdischarge mortality rates among HNHC patients of 8.9% and 17.7%, respectively [16, 17].

H3 and HaH share certain important features. The H3 model, like HaH programs, is designed to provide hospital‐level care in the home. In addition, as with HaH programs, admission avoidance accounts for the vast majority of associated reductions in health care expenditures in the H3 model [10, 18]. However, H3 differs from traditional HaH programs in important ways. For example, while HaH is focused on managing patients through acute care episodes, H3 is designed to manage patients intensively at home for several weeks to months; this focus on longer term management necessitates a dual focus on both managing acute illnesses—which commonly arise in high‐need, high‐cost patients, and a focus on whole‐person health and condition management. Furthermore, most HaH models are designed to treat populations with SCCM Level 0 care needs—hospitalized medical and surgical patients with no intensive monitoring or care requirements [19]. In contrast, the majority of H3 patients had SCCM Levels 1–2 care needs—patients who have a single failing organ system, have a real risk of experiencing clinical deterioration, and/or who require continuous telemetry for some portion of their care episode—and would have been excluded from traditional HaH enrollment criteria [20, 21, 22]. Finally, unlike HaH programs, which traditionally manage a variety of different conditions in the home, H3's focus is on patients with cardiovascular comorbidities. This focus, and the heightened risk for high‐acuity cardiovascular events and care needs in the H3 population, has led H3 to evolve differently from HaH to effectively address the specific care gaps and needs of this population.

This study has important limitations. First, the retrospective nature of this study limits our ability to infer causal links between the H3 intervention and observed outcomes. Our findings should be considered hypothesis‐generating, and a randomized controlled trial is needed to confirm them. Second, and in building on the first limitation, while objective referral criteria for H3 were established, it is possible that referring PCPs and emergency selected a subset of patients who met these criteria and who they thought were most likely to benefit from the H3 intervention. It is therefore possible that a subset of HNHC patients might benefit more from H3 than others, and future studies should focus on better understanding which populations of patients stand to derive greatest benefits from H3 and interventions like it. Third, the H3 intervention was studied in urban and suburban regions of Southeast Florida, and results may not be generalizable to different geographic regions—particularly rural areas with longer drive times to patients' homes or inconsistent internet availability. Future work should attempt to elucidate the degree to which certain geographic and community characteristics impact H3 outcomes. Fourth, we cannot rule out the possibility that regression to the mean accounted for some of the observed reductions in hospitalizations and costs associated with enrollment in H3.

In summary, in a population of high‐need, high‐cost patients with cardiovascular comorbidities, enrollment in a longitudinal intensive home‐based care model which combined in‐person care, virtual care, and intensive cardiovascular remote monitoring was associated with significant reductions in hospitalization rates, inpatient days, and total and part A costs of care.

## Authors Contributions

Michael Shen was responsible for the conception, enrollment, and medical treatment for the study, and contributed to revisions of the final version of the manuscript. Kareem Osman performed analysis and interpretation of the data and wrote the initial draft of the manuscript. Daniel Blumenthal performed analysis of the data and contributed to revisions of the final version of the manuscript. Kaelin DeMuth performed analysis of data and contributed to revisions of the manuscript. Yixiang Liu was responsible for the conception of the study.

## Conflicts of Interest

Dr. Shen reports serving as the Chief Medical Officer for Novolink Health, which is a Division of Cardiovascular Associates of America. Dr. Blumenthal reports serving as the Chief Quality Officer for Cardiovascular Associates of America and the President of Novocardia, which is a Division of Cardiovascular Associates of America.

## Supporting information

Supporting information.

Supporting information.

Supporting information.

Supporting information.

## Data Availability

Data sets generated during the current study are available from the corresponding author on reasonable request.
