# Supplementary figures and images for: Home Heart Hospital Associated With Reduced Hospitalizations and Costs Among High‐Cost Patients With Cardiovascular Disease
Source: Clin Cardiol. 2024 Jun 14;47(6):e24302. doi: 10.1002/clc.24302 (PMC11177177; doi:10.1002/clc.24302)

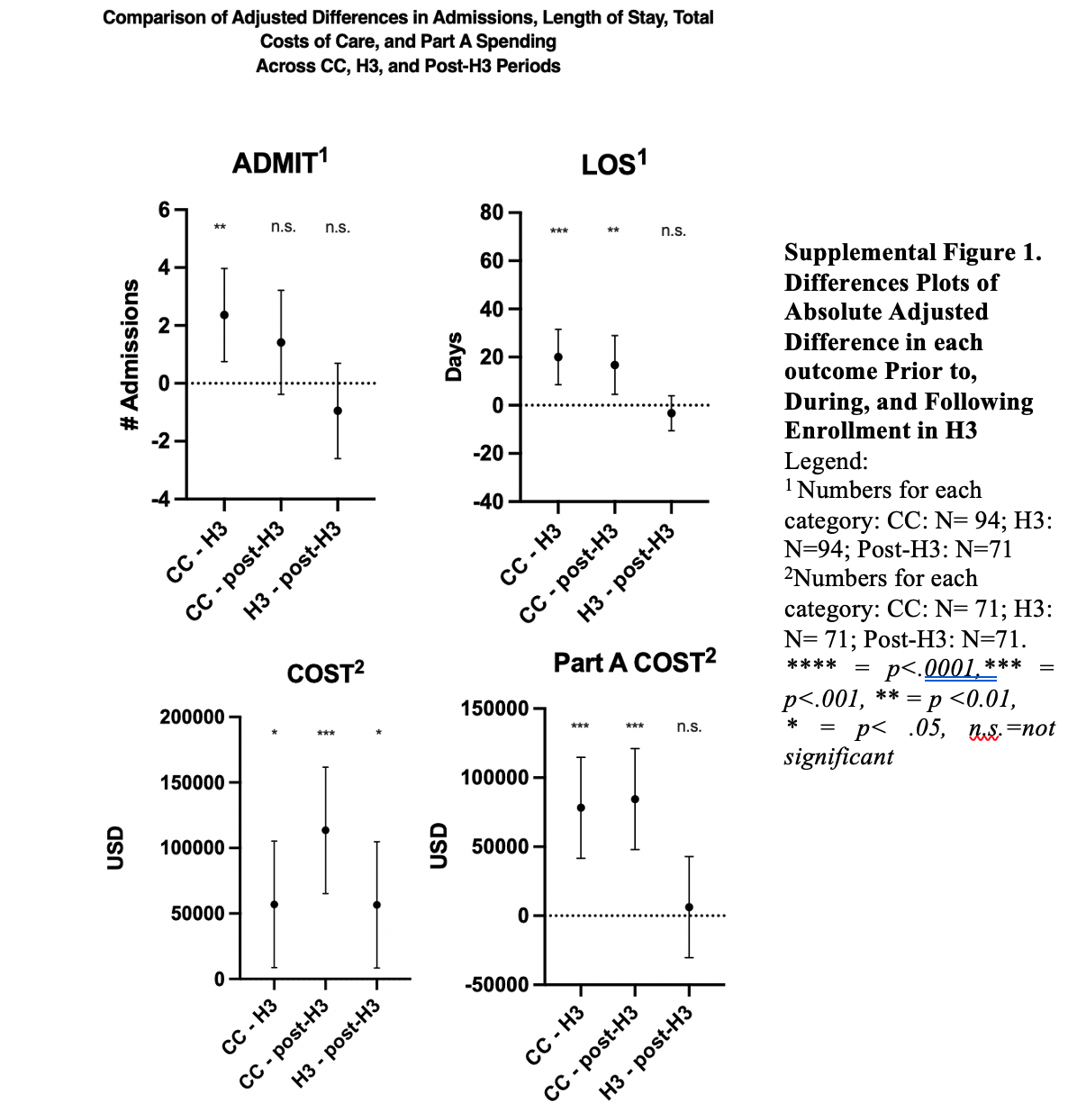

Supplement: Supplementary file 1 — Supporting information. [file CLC-47-e24302-s002.png]

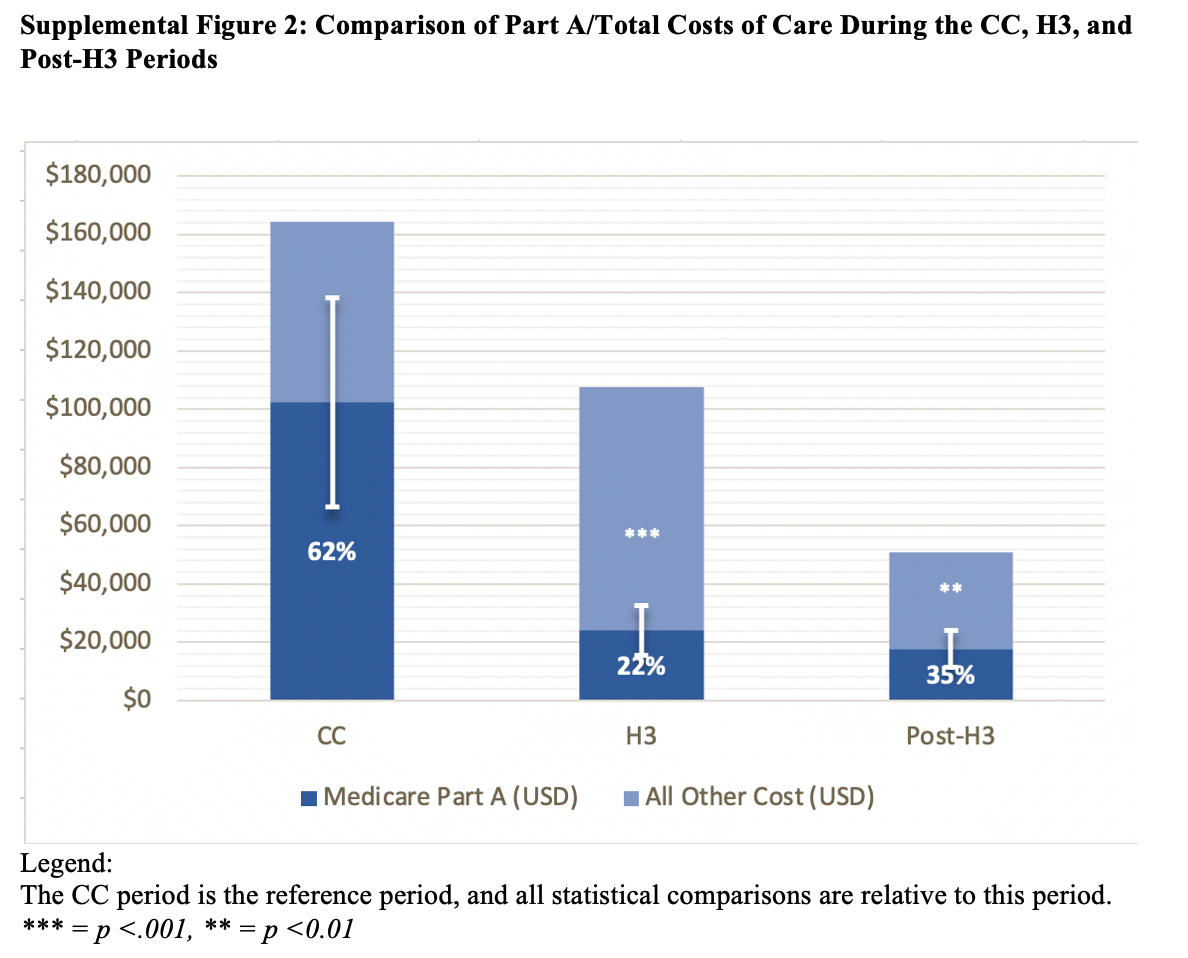

Supplement: Supplementary file 2 — Supporting information. [file CLC-47-e24302-s003.png]
