# Supplementary material for: Home Heart Hospital Associated With Reduced Hospitalizations and Costs Among High‐Cost Patients With Cardiovascular Disease
Source: Clin Cardiol. 2024 Jun 14;47(6):e24302. doi: 10.1002/clc.24302 (PMC11177177; doi:10.1002/clc.24302)
Supplement: Supplementary file 3 — Supporting information. [file CLC-47-e24302-s004.docx]

**Supplemental Table 1. Level of Care Distribution and Outcomes during H3 Enrollment**

|  | **SCCM 0 (n=35)** | **SCCM 1 (n=54)** | **SCCM 2 (n=5)** |
| --- | --- | --- | --- |
| **Average days enrolled in H3 (95% CI)** | 71 (58 - 85) | 80 (49-110) | 259 (15 -303)* |
| **Mean Annualized Admissions per Person (95% CI)** | 1.74 (0.42-3.07) | 2.50 (0.69-4.32) | 1.77 (-0.35-3.9) |
| **Mortality—N (%)** | 1 (2.8%) | 4 (7.4%) | 0 (0%) |

**Legend:**

The SCCM 0 group period is the reference period. All statistical comparisons are relative to this period.

** = p <0.05*
