# Supplementary material for: Home Heart Hospital Associated With Reduced Hospitalizations and Costs Among High‐Cost Patients With Cardiovascular Disease
Source: Clin Cardiol. 2024 Jun 14;47(6):e24302. doi: 10.1002/clc.24302 (PMC11177177; doi:10.1002/clc.24302)
Supplement: Supplementary file 4 — Supporting information. [file CLC-47-e24302-s001.docx]

**Supplemental Table 2. Annualized Admissions, Inpatient Days, and Health Care Costs Prior to, During, and Following Enrollment in H3 for 71 Patients**

|  | **CC** | **H3** | **30 Days Post-H3** | **90 Days Post-H3** |  | **180 Days Post-H3** |
| --- | --- | --- | --- | --- | --- | --- |
| **Mean annualized admissions per person (95% CI)^1^** | 4.4 (3.1-5.6) | 2.0 (0.7-3.4)* | 0.5 (0.2-0.8)*** | 0.8 (0.4-1.6)*** |  | 2.8 (1.8-3.9) |
| **Mean annualized number of inpatient days per person (95% CI)^1^** | 28.9 (18.0-39.9) | 8.5 (2.3-14.8)*** | 2.5 (1.3-3.8)*** | 4.8 (1.2-3.6)*** |  | 10.6 (5.7-15.5)* |

**Legend:**

Values are n (95% CI) or n (% of total)

^1^ Numbers for each period: CC: N= 71; H3: N=71; Post-H3: N=71

The CC period is the reference period, and all statistical comparisons are relative to this period.

The mixed effects model included both random effects and fixed effects, including missing data, intersubject variability, and time period.

**** = p <0.001, ** = p <0.01, * = p < 0.05*
